# Nanovesicles from *Rosa canina*: A Treasure Trove of Antioxidant Potential for Oxidative Stress, Inflammation, and Gut Microbiota Modulation

**DOI:** 10.3390/ph18111672

**Published:** 2025-11-05

**Authors:** Gaia Cusumano, Agnese Bertoldi, Eleonora Calzoni, Husam B. R. Alabed, Laura Dorina Dinu, Emanuel Vamanu, Florentina Matei, Gokhan Zengin, Carla Emiliani

**Affiliations:** 1 Department of Chemistry, Biology and Biotechnology, University of Perugia, 06100 Perugia, Italy; gaia.cusumano@dottorandi.unipg.it (G.C.); agnese.bertoldi@dottorandi.unipg.it (A.B.); husambr.alabed@unipg.it (H.B.R.A.); carla.emiliani@unipg.it (C.E.); 2 Faculty of Biotechnologies, University of Agricultural Sciences and Veterinary Medicine, 011464 Bucharest, Romania; laura.dinu@biotehnologii.usamv.ro (L.D.D.); emanuel.vamanu@gmail.com (E.V.); florentina.matei@biotehnologii.usamv.ro (F.M.); 3 Faculty of Food Industry and Tourism, Transilvania University of Brasov, 500015 Brasov, Romania; 4 Department of Biology, Science Faculty, Selcuk University, Konya 42130, Turkey; gokhanzengin@selcuk.edu.tr; 5 Centro di Eccellenza Materiali Innovativi Nanostrutturati (CEMIN), University of Perugia, Via del Giochetto, 06123 Perugia, Italy

**Keywords:** plant-derived extracellular vesicles, *Rosa canina*, extracellular vesicles, anti-inflammatory activity, antioxidant activity, gut microbiota

## Abstract

**Background/Objectives:** Extracellular vesicles (EVs) have become key facilitators of communication between cells, significantly influencing various physiological functions. Although EVs originating from mammalian cells have been heavily researched for their therapeutic applications, there is a growing interest in extracellular vesicles derived from edible plants (PDEVs) because of their unique bioactive characteristics. These nanovesicles (NVs) exhibit remarkable biocompatibility, low immunogenicity, and the ability to overcome biological barriers, making them promising candidates for biomedical applications. This study aimed to evaluate the antioxidant and anti-inflammatory properties of NVs isolated from *Rosa canina* berries. **Methods:** Antioxidant activity was assessed through in vitro assays, confirming their ability to fight oxidative stress. Additionally, enzymatic inhibition tests were conducted to explore their potential role in regulating key metabolic pathways associated with inflammation and oxidative damage. The antioxidant and anti-inflammatory activity of *Rosa canina* NVs was further tested on a THP-1 cell-based inflammation model, demonstrating their ability to modulate the inflammatory response at the cellular level. Moreover, the impact of these NVs on gut microbiota was investigated to assess their protective effects on antibiotic-induced dysbiosis. **Results:** The results demonstrated their ability to modulate oxidative stress, regulate enzymatic pathways, reduce inflammation in THP-1 cells, and influence gut microbiota in a positive manner.

## 1. Introduction

Inflammation is a complex physiological response aimed at restoring tissue homeostasis following damage, infection, or stress. Although essential in the body’s defense mechanisms, when chronic or not adequately regulated, it becomes a powerful promoter of multiple pathologies. Persistent inflammation is in fact implicated in the physiopathology of cardiovascular, neurodegenerative, oncological, autoimmune, and metabolic diseases, assuming a pivotal role in the process of chronicization [1,2]. This condition is marked by oxidative stress, which occurs when the reactive oxygen species (ROS) exceed the organism’s antioxidant capabilities, leading to increased harm via lipid peroxidation, alterations in proteins and nucleic acids, and the triggering of inflammatory pathways [3,4,5]. In this scenario, a new class of biological nanocarriers has gained increasing interest: plant-derived extracellular vesicles (PDEVs). These bilayer lipid membrane-enhanced nanovesicles are secreted by plant cells and contain a heterogeneous molecular cargo such as messenger RNAs and microRNAs, proteins, lipids, secondary metabolites, and phenolic compounds, capable of exerting complex biological activities in animal and human systems [6,7,8]. The distinctive feature of PDEVs is their origin from edible plants, such as ginger, carrot, cabbage, green tea, and grapes, which allows safe oral administration and facilitates their accessibility and sustainability [9,10,11,12]. Isolated from food matrices through ultracentrifugation methods or more advanced purification techniques, PDEVs have demonstrated an extraordinary ability to cross biological barriers such as the intestinal or blood–brain barrier, maintaining the structural integrity and functionality of their content [13,14]. A lot of studies have confirmed that PDEVs are not only biocompatible and poorly immunogenic, but also exert antioxidant, anti-inflammatory, antitumor, and immunomodulatory activities [15,16,17,18,19,20,21]. For example, PDEVs derived from green tea and ginger have shown the ability to reduce the production of pro-inflammatory cytokines (TNF-α, IL-6) and inhibit the activation of the NLRP3 inflammatory complex, acting in murine models of colitis [22,23]. At the same time, PDEVs can positively modulate the intestinal microbiota, contributing to the maintenance of digestive and systemic homeostasis [24,25,26,27]. Furthermore, thanks to their potential to remodel the microbial composition, PDEVs can prevent or correct intestinal dysbiosis, a condition characterized by imbalances in the bacterial community associated with chronic inflammation and gastrointestinal pathologies, by supporting the restoration of the mucosal barrier and microbial diversity [28,29,30]. Among the edible plants of phytotherapeutic interest, *Rosa canina* represents a rich source of bioactive compounds, such as vitamin C, flavonoids, phenolic acids, and carotenoids, that have always been used in traditional medicine for the treatment of inflammatory conditions, respiratory tract infections, and gastrointestinal disorders [31,32,33,34,35]. Despite the growing interest in PDEVs for their biocompatibility and therapeutic potential, *Rosa canina* has not yet been explored as a source of extracellular vesicles. This species is particularly rich in polyphenols and antioxidant compounds, suggesting that its nanovesicles may carry unique molecular cargoes with potential anti-inflammatory and cytoprotective properties distinct from those of vesicles derived from other edible plants [31]. Preliminary studies suggest that EVs derived from plants with high phenolic content can selectively transport bioactive molecules with targeted action on inflammatory and oxidative pathways [7,15,36,37,38,39]. The present study aims to investigate the functional characteristics of nanovesicles obtained from *Rosa canina* berries, with particular attention to their antioxidant and anti-inflammatory potential. Through the use of in vitro assays, inflammatory cell models based on THP-1 lines, and evaluations on the intestinal microbiota, this work aims to provide new evidence on the possible use of *Rosa canina* NVs as multifunctional tools for the modulation of oxidative stress, inflammatory response, and intestinal homeostasis, promoting the development of natural and sustainable therapeutic strategies.

## 2. Results

### 2.1. Rosa canina Nanovesicles Characterization: NTA and SEM Analysis

**Figure 1:**
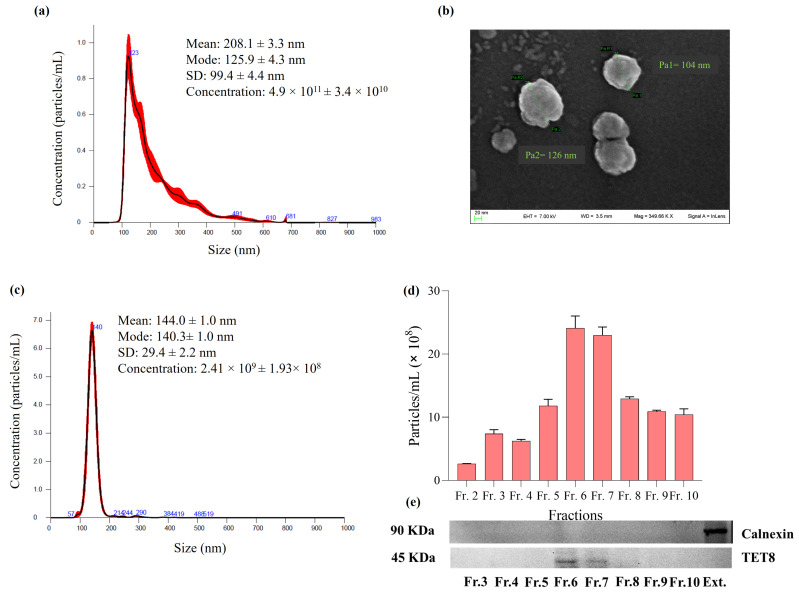
NTA (**a**), SEM images (**b**), size distribution of the most enriched fraction 6 analyzed through NTA (**c**), particle concentration of different density gradient fractions reported as Particles/mL obtained by NTA (**d**), TET8 and Calnexin detection in gradient fractions (from 3 to 10) and *R. canina* extract (Ext) obtained by immunoblotting (**e**). Data are expressed as mean ± SD (n = 3).

*R. canina* NVs were characterized for morphology, size distribution, and membrane charge by SEM, NTA, and DLS analyses (Figure 1) [40,41]. The NTA revealed that *R. canina* NVs were characterized by an average diameter of 208.1 ± 3.3 nm, with the most frequently occurring size (mode) being 125.9 ± 4.3 nm. The isolation procedure yielded 4.9 × 10^11^ particles per mL, corresponding to an estimated yield of about 9.8 × 10^10^ vesicles per gram of berries. Moreover, the total protein content associated with the vesicle preparation was approximately 35 µg per gram of berries. To gain more detailed insight into their morphology, scanning electron microscopy (SEM) was conducted (Figure 1b). According to the SEM image, *R. canina* NVs showed the presence of spherical vesicles with mean diameters between 100 and 130 nm and a slightly rough surface texture, confirming the typical features of EVs. Moreover, *R. canina* NVs Zeta-potential resulted in negative values (−7.93 mV) (Figure S2). These values are comparable to the Zeta-potential determined by the surface charge of other PDEVs. The Zeta-potential reflects the overall surface charge of NVs and provides an indirect measure of their colloidal stability, which governs both vesicle–vesicle and vesicle–medium interactions. In general, ZP values close to neutrality indicate reduced electrostatic repulsion and, consequently, a higher propensity for aggregation [42,43].

**Figure 2:**
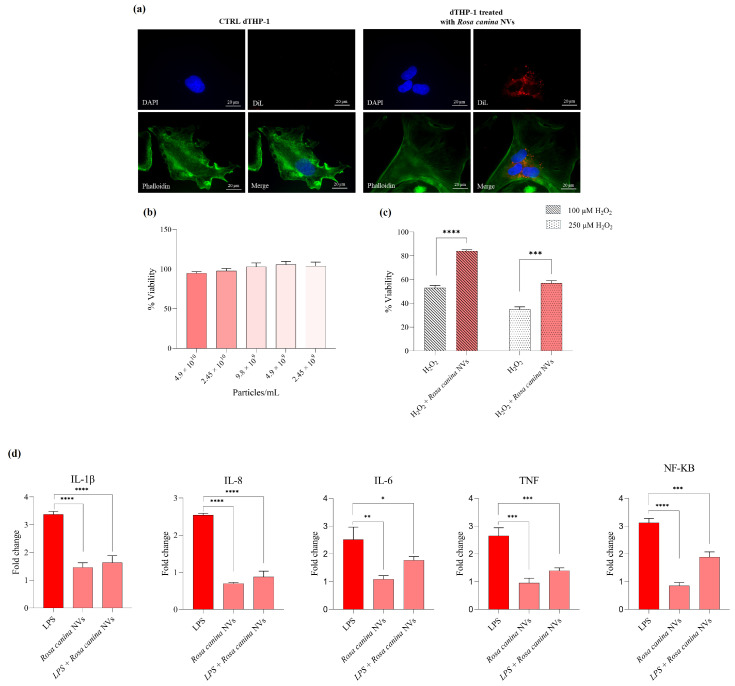
DiL-labeled-NVs internalization by differentiated THP-1 cells (dTHP-1) with the nuclei marker DAPI (blue, DAPI filter), along with relative phalloidin and merged images (image magnification: 60×). In the left panel were reported control cells treated with extraction buffer processed through the same differential centrifugation and DiL-labeling procedure; in the right panel were reported dTHP-1 cells incubated with DiL-labeled NVs showing intracellular fluorescence (**a**). MTT viability assay (**b**), antioxidant activity (**c**), and anti-inflammatory activity of *R. canina* NVs (**d**). Data are expressed as mean ± SD (n = 3). Significance is indicated as follows: * *p* < 0.05, ** *p* < 0.01, *** *p* < 0.001, **** *p* < 0.0001.

Considering the need to obtain a highly pure vesicle population, *R. canina* NVs were further purified by density gradient ultracentrifugation on an iodixanol gradient. Ten gradient fractions were collected and analyzed by NTA (Figure S1) to quantify particle concentration and assess size distribution. Among these, fraction 6 exhibited the highest vesicle concentration (2.41 × 10^9^) and displayed a monodisperse profile centered at approximately 140 nm (Figure 2c,d), consistent with the expected size range of plant-derived extracellular vesicles. To confirm vesicle enrichment after density gradient separation, immunoblotting for the PDEV marker TET8 was performed on the collected fractions (Figure 1e). In parallel, a crude extract from *R. canina* was analyzed as a control. A clear positive signal for TET8 was detected in fractions 6 and 7, corroborating the NTA results in terms of both particle concentration and size homogeneity. Moreover, calnexin, an endoplasmic reticulum protein commonly used as a negative marker for extracellular vesicles, was detected only in the crude extract and not in the purified NV fraction. This result further supports the high purity of the isolated vesicles. The combination of NTA, SEM, DLS, and immunoblotting confirms the vesicular nature of the isolated *R. canina* NVs and demonstrates that the isolation yields a clean, homogeneous, vesicle-enriched population.

### 2.2. Untargeted Polyphenols Analysis Through LC/MS

In order to investigate the presence of polyphenols within the nanovesicles isolated from *R. canina*, a liquid chromatography–mass spectrometry (LC-MS) analysis was conducted. In parallel, a semi-quantitative assessment of phenolic compounds was carried out, using the total polyphenol content (TPC), previously determined by the Folin method, as a reference [44]. This combined approach enabled the identification and quantification of different bioactive molecules, predominantly polyphenols, as reported in Table 1.

NVs isolated from *R. canina* have shown a rich metabolomic profile characterized by the presence of interesting bioactive compounds, with a predominance of polyphenols and flavonoids known for their antioxidant, anti-inflammatory, and protective properties. LC-MS analysis revealed that the most abundant metabolite was (-)-epicatechin, in a concentration of 6.37 ± 0.42 µg per gram of starting berries, followed by hederagenin glucoside and phloretin-2′-*O*-glucoside, both with about 2 µg/g, and by quercetin-3-*O*-rutinoside with 1.95 ± 0.36 µg/g. These compounds, belonging to the flavonoid class, are recognized for their scavenger activity against free radicals and for the potential beneficial effect on chronic diseases related to oxidative stress. The presence of gallic acid, equal to 1.10 ± 0.24 µg/g, was also highlighted, together with its derivatives such as methyl gallate and gallic acid hexoside, which further confirm the antioxidant potential of NVs. Molecules such as naringenin, eriocitrin, and proanthocyanidins B1 and C1, albeit in lower concentrations, contribute to the variety and complexity of the phenolic profile. Less represented but pharmacologically interesting bioactive compounds were also identified, including tormentic acid, rhein, salicylic acid, and corosolic acid, suggesting that *R. canina* NVs may convey a plethora of metabolites with potential therapeutic effects.

### 2.3. Rosa canina Nanovesicles Antioxidant Activity

The assessment of antioxidant activity in plant-derived products is a crucial step in identifying bioactive molecules with therapeutic potential, particularly for the prevention and treatment of oxidative stress-related diseases, which are frequently associated with chronic pathologies [45]. In the present study, the antioxidant properties of *R. canina*-derived NVs were investigated through a series of in vitro assays, including DPPH (2,2-Diphenyl-1-picrylhydrazyl), ABTS (2,2′-Azino-bis(3-ethylbenzothiazoline-6-sulfonic acid)), CUPRAC (cupric ion reducing antioxidant capacity), and FRAP (ferric reducing antioxidant power). These complementary analytical methods were employed to evaluate both the radical scavenging potential and the reducing capacity of the vesicles toward transition metal ions. The integration of different assay systems allowed for a more comprehensive characterization of the antioxidant behavior of *R. canina* NVs, supporting their potential application in the development of bioactive compounds derived from plant sources with possible relevance in preventing oxidative stress-related disorders. Table 2 reports the results of the antioxidant capacity of *R. canina*-derived NVs.

*R. canina* NVs showed a total phenolic content (TPC) of 61.16 ± 4.36 mg gallic acid equivalents (GAEs) per gram of starting berries, indicating a significant presence of phenolic compounds, known for their antioxidant properties. The total flavonoid content (TFC), equal to 2.69 ± 0.18 mg rutin equivalent (RE) per gram, suggests that flavonoids represent only a small part of the total phenolic compounds. The antioxidant activity was investigated through DPPH and ABTS assays, which revealed a good scavenger capacity with values of 56.57 ± 4.44 mg and 76.32 ± 3.58 mg Trolox equivalents (TEs) per gram, respectively. These results indicate an effective neutralizing activity against free radicals in both lipophilic (DPPH) and hydrophilic (ABTS) environments. In parallel, the FRAP and CUPRAC assays showed a marked capacity to reduce metal ions, with values of 56.10 ± 1.48 mg TE/particles and 97.48 ± 1.17 mg TE/particles, respectively. In particular, the high value obtained in the CUPRAC test underlines the strong overall antioxidant activity of NVs. Finally, the chelating capacity analysis showed a value of 24.53 ± 3.70 mg EDTA equivalents (EDTAEs) per gram, indicating a significant ability to sequester pro-oxidant metal ions. This function further contributes to the protection against oxidative stress, preventing the formation of metal-mediated reactive oxygen species (ROS).

In addition to antioxidant properties, *R. canina* NVs were evaluated for their enzyme inhibitory activity in skin disorders, inflammation, diabetes, and hyperglycemia. In this context, in the present study, the inhibitory potential of nanovesicles (NVs) isolated from *R. canina* against several target enzymes, including acetylcholinesterase (AChE), butyrylcholinesterase (BChE), tyrosinase, α-amylase, and α-glucosidase, was investigated. The results obtained, expressed as equivalents of the respective pharmacological standards, are reported in Table 3.

The inhibition of acetylcholinesterase (AChE) was found to be equal to 0.65 ± 0.08 mg galantamine equivalents (GALAEs) per particle, suggesting a moderate activity in the potential maintenance of synaptic acetylcholine levels. The inhibition of butyrylcholinesterase (BChE) was more marked, with a value of 3.50 ± 0.05 mg GALAE/particles, highlighting a potential efficacy of NVs in the more advanced stages of neurodegenerative diseases, such as Alzheimer’s disease, where BChE activity tends to increase.

Regarding the inhibition of tyrosinase, a key enzyme in melanin synthesis, a value of 59.56 ± 3.35 mg kojic acid equivalents (KAEs) per particle was observed. This data indicates a significant inhibitory capacity, supporting the potential application of NVs for the treatment of skin disorders. Finally, the inhibitory activity towards enzymes involved in carbohydrate metabolism was evaluated by the assays on α-amylase and α-glucosidase. The results showed an inhibition of 0.22 ± 0.03 and 1.21 ± 0.02 mmol acarbose equivalents (ACAEs) per particle, respectively. In particular, the high value detected for α-glucosidase suggests an interesting hypoglycemic activity of NVs, indicative of a potential use in post-prandial glycemic control, and therefore, in the management of type 2 diabetes.

### 2.4. Intracellular Antioxidant and Anti-Inflammatory Potential of Rosa canina NVs

To evaluate the antioxidant and anti-inflammatory potential of NVs isolated from *R. canina*, in vitro studies were conducted to assess their cytotoxicity, antioxidant activity under conditions of induced oxidative stress, and their ability to modulate the production of selected pro-inflammatory cytokines (Figure 2). Moreover, to confirm the internalization of *R. canina* NVs into differentiated THP-1 monocytes, a fluorescence microscopy analysis was first performed following incubation with DiL-labeled-NVs. The microscopy images revealed a distinct intracellular red fluorescence signal, indicating that the vesicles were efficiently taken up by the cells rather than remaining bound to the cell surface (Figure 2a).

As shown in Figure 2b, THP-1 cells were treated with increasing concentrations of *R. canina* NVs (ranging from 2.45 × 10^9^ to 4.9 × 10^10^ particles/mL) for 24 h. No significant changes in cell viability were observed compared to untreated controls, indicating that *R. canina* NVs are well tolerated by THP-1 cells across the tested concentrations, with viability consistently exceeding 90%.

Based on these results, the potential protective effects of *R. canina* NVs under oxidative stress conditions were subsequently evaluated. To this end, THP-1 cells were exposed to hydrogen peroxide (H_2_O_2_) at 100 µM and 250 µM, with or without co-treatment with *R. canina* NVs (2.45 × 10^10^ particles/mL). As illustrated in Figure 2c, H_2_O_2_ treatment alone significantly reduced cell viability. However, co-treatment with *R. canina* NVs notably mitigated this cytotoxic effect at both concentrations; in fact, cell viability was significantly higher in the H_2_O_2_ + *R. canina* NVs groups compared to H_2_O_2_ alone (**** *p* < 0.0001 for 100 µM; *** *p* < 0.001 for 250 µM), supporting a protective antioxidant role for *R. canina* NVs in this cellular model.

To further explore the bioactivity of *R. canina* NVs, their potential anti-inflammatory effects were evaluated by measuring the expression levels of the pro-inflammatory mediators including the pro-inflammatory cytokines IL-1β, IL-6, and IL-8, the tumor necrosis factor TNF, and the transcription factor NF-κB, following lipopolysaccharide (LPS) stimulation. As shown in Figure 2d, LPS treatment induced a significant upregulation of all analyzed genes, confirming the activation of a strong inflammatory response. On the contrary, pre-treatment with *R. canina* NVs (2.45 × 10^10^ particles/mL), either alone or in combination with LPS, resulted in a marked reduction in IL-1β, IL-6, and IL-8, indicating an effective inhibition of interleukin-mediated inflammatory signaling (*p* < 0.0001 or *p* < 0.05). A similar trend was observed for TNF and NF-κB, whose expression was significantly decreased in NV-treated groups compared to LPS-stimulated cells (*p* < 0.001). Given the central role of NF-κB as a transcriptional regulator of cytokine expression, its downregulation suggests that *R. canina* NVs may exert their anti-inflammatory activity by modulating upstream transcriptional pathways.

### 2.5. In Vitro Effect of Rosa canina NVs on Antibiotic-Induced Dysbiosis

To investigate the ability of *R. canina* NVs to minimize the kanamycin-induced perturbation of the human gut microbiota, the in vitro gastro intestinal simulation system (GIS1) was used [46]. The gut microbiota has been previously analyzed and tested in different studies [47]. At the same time, sterile distilled water (M treatment), kanamycin (MKm treatment), *R. canina* NVs (MEVs treatment), and the co-administration of NVs and antibiotics (MKmEVs treatment) were added daily during the 5-day experiments. At the end of the experiment, the total microbial load (aerobic and anaerobic counts) on non-selective media and the beneficial bacterial (lactic acid bacteria, bifidobacteria) growth on specific selective media were evaluated. Moreover, qPCR analysis was used to assess the effect of different treatments on two major phyla Firmicutes (F) and Bacteroidetes (B) from gut microbiota using the F/B quantitative ratio and to quantify both beneficial bacteria, *Lactobacillus* spp. and *Bifidobacterium* spp. These major phyla include almost 90% of microbial species inhabiting the human gut, while other less abundant but still significant phyla include Actinobacteria, Proteobacteria, Fusobacteria, and Verrucomicrobia [48]. The phylum F/B ratio is an important indicator of gut microbiota diversity and overall gut health, while the quantitative F/B ratio provides numerical information about the effect of stress on different intestinal microbiota groups [48].

**Figure 3:**
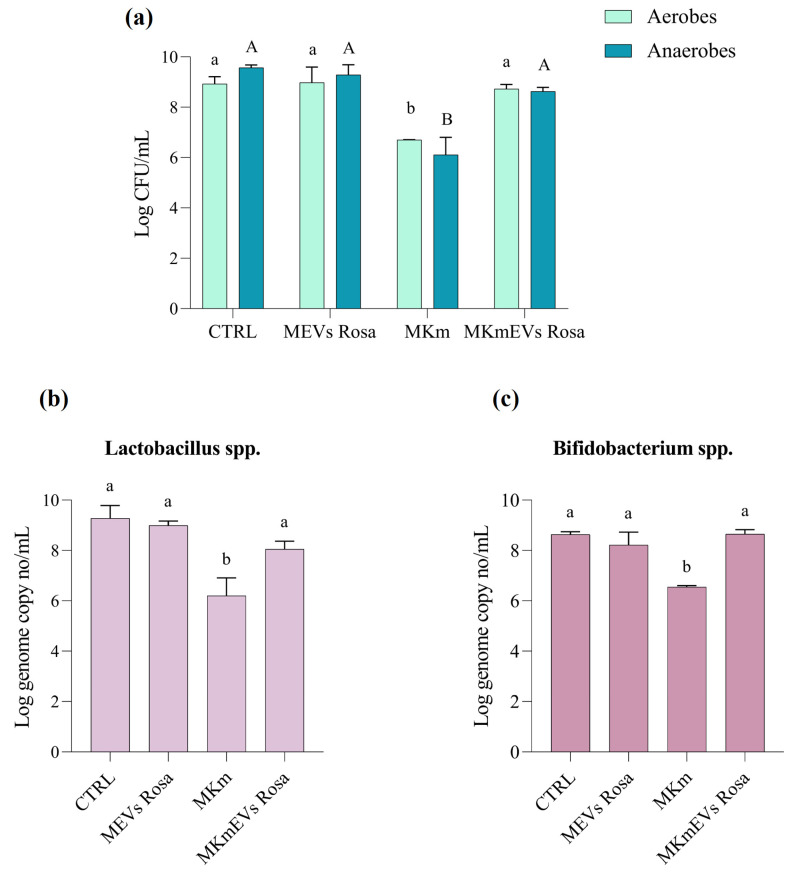
The effect of the *R. canina* NVs on the total microbial load detected on non-selective media (**a**), *Lactobacillus* spp. (**b**), and *Bifidobacterium* spp. (**c**) detected by qPCR, in different experiments: control, MEVs Rosa with administration of *R. canina* NVs, MKm with kanamycin treatment, and MKmEVs Rosa with co-administration of *R. canina* NVs and kanamycin. Data are expressed as mean ± SD (n = 3). Columns labeled with different letters are significantly different at *p* < 0.0001.

Our data showed that bacterial counts in samples treated with antibiotics decreased by 2.2 log CFU/mL for aerobes and by 3.45 log CFU/mL for anaerobes, compared to the control (M) experiment. However, co-administration of kanamycin and NVs decreased the microbial load by log 1.13 CFU/mL compared to the M experiment, suggesting the protective role of *R. canina* NVs on gut bacterial richness in stress conditions. In both experiments with antibiotic treatments, the total number of culturable germs decreased, but the total counts after NVs-kanamycin administration were approximately 4.54 log CFU/mL higher than in the case of solely antibiotic administration. Additionally, NV treatment did not significantly change the total microbial load. A lower decrease was noted for the aerobic population, from 8.92 ± 0.28 log CFU/mL in the control experiment to 6.70 ± 0.005 log CFU/mL and 8.73 ± 0.17 log CFU/mL after kanamycin and kanamycin-NVs treatments, respectively (Figure 3a).

It is known that kanamycin treatment can decrease the abundance of beneficial bacteria like Lactobacillus and Bifidobacterium, while potentially promoting the growth of other bacteria like Enterococcus and Clostridium [49]. Still, some Lactobacillus strains proved intrinsic resistance to kanamycin [50]. In our experiments, the antibiotic treatment decreased the number of both beneficial lactic acid bacteria and bifidobacteria based on the microbiological data, which were confirmed by qPCR results (Figure 3b). The initial density of *Lactobacillus* spp. measured by the qPCR was 9.27 ± 0.50 log/mL and it decreased to 6.20 ± 0.71 log/mL and 8.05 ± 0.32 log/mL after MKm and MKmEVs treatments, respectively (Figure 3b). However, the population of *Bifidobacterium* spp. exceeded 8.63 ± 0.11 log/mL in the control experiment, and they were recovered mainly by co-administration of antibiotics and NVs, while the population reduced by 2.08 log/mL after kanamycin treatment (Figure 3c). Surprisingly, both populations of beneficial bacteria slowly decreased in the experiment with *R. canina* NVs treatment, especially lactobacilli.

Based on qPCR results with specific primers, the quantitative F/B ratio clearly decreased (F/B = 2.3) in the experiment with antibiotic-treated gut microbiota, compared to the rest of the experiments (Table 4). That result proved that the kanamycin administration induced gut microbiota imbalance and affected both major phyla. Although co-administration of antibiotics and *R. canina* NVs reduced the qPCR counts for Firmicutes and Bacteroidetes, the quantitative F/B ratio was maintained (F/B = 3.2), suggesting the ability of *R. canina* NVs to minimize the kanamycin-induced perturbation of the human gut microbiota.

## 3. Discussion

This study provides a comprehensive characterization of nanovesicles (NVs) isolated from *R. canina*, confirming their structural integrity, biochemical composition, and biological potential. To our knowledge, this is the first study describing the isolation and biological activity of nanovesicles derived from *Rosa canina*. Compared to vesicles from other previously characterized edible plants, *R. canina* NVs exhibit a distinctive bioactive profile likely related to their enrichment in antioxidant phenolic compounds, which may account for their potent anti-inflammatory effects and high stability in biological systems. To gain detailed insights into *R. canina*-derived EVs, several characterization techniques were combined, including size distribution assessments by NTA on both the enriched population and the fractions obtained after density gradient separation, Z-potential analysis, and morphology analysis by SEM. Nanoparticle Tracking Analysis (NTA) revealed that *R. canina* NVs had an average diameter of 208.1 ± 3.3 nm and a mode size of approximately 125.9 ± 4.3 nm (Figure 1a) for the enriched fraction and 144.0 ± 1.0 nm for fraction 6 obtained after gradient purification. Scanning electron microscopy (SEM) images further showed spherical vesicles with sizes ranging from 100 to 130 nm and slightly rough surfaces (Figure 1b). The evaluation of surface charge revealed that *R. canina* NVs exhibited a negative Zeta-potential. The average values were quantified as −7.93 mV. The negative surface charge indicates their potential to interact with positively charged molecules or surfaces, thereby facilitating specific cellular uptake or adhesion events. Previous studies have documented the presence of negatively charged EVs in plant-derived samples [51,52]. Moreover, the negative Zeta-potential observed for *R. canina* NVs could arise from the composition of their membrane lipids and associated surface molecules. In particular, the presence of negatively charged phospholipids (such as phosphatidylserine and phosphatidic acid), while neutral lipids (e.g., phosphatidylcholine) can attenuate it, resulting in the moderate stability typically observed for plant-derived vesicles. The consistency between NTA measurements and morphological observations supports the identification of these structures as plant-derived extracellular vesicle-like structures. Moreover, immunoblotting analysis shows that TET8, a known marker of plant extracellular vesicles, is specifically detected only in the vesicle-enriched fractions (fractions 6 and 7) obtained via density gradient ultracentrifugation. In contrast, calnexin, an endoplasmic reticulum marker, is present only in the total *R. canina* extract and is absent from the vesicle fractions. These results confirm the selective enrichment of vesicular components in the isolated fractions and demonstrate the effective separation of NVs from intracellular contaminants, supporting the vesicular identity and purity of the preparation. LC-MS metabolomic characterization demonstrated that the NVs are enriched in several classes of bioactive metabolites, particularly polyphenols and flavonoids, which contribute to their potential antioxidant and anti-inflammatory activities. The most abundant metabolite, (-)-epicatechin (6.37 ± 0.42 µg/particles), is especially noteworthy due to its established role in neutralizing free radicals and modulating inflammatory responses [53,54,55,56]. Hederagenin glucoside and phloretin-2′-*O*-glucoside were also present at approximately 2 µg/particles, while quercetin-3-*O*-rutinoside was quantified at 1.95 ± 0.36 µg/particles, further contributing to the complex phenolic profile. The presence of gallic acid (1.10 ± 0.24 µg/particles), together with its derivatives such as methyl gallate and gallic acid hexoside, reinforces the antioxidant potential of the NVs, given the documented roles of these compounds in oxidative stress reduction and inflammation modulation [57,58]. Additionally, molecules such as naringenin, eriocitrin, and procyanidins B1 and C1, although present in lower concentrations, enrich the phenolic profile, suggesting possible synergistic interactions among multiple bioactive metabolites [59,60,61,62]. Among the less abundant but pharmacologically relevant compounds identified were tormentic acid, rhein, salicylic acid, and corosolic acid, all of which have been associated with anti-inflammatory, anti-aging, and metabolic regulatory effects [63,64]. Compared to nanovesicles from other edible plants such as ginger, *Citrus limon*, *Camellia sinensis*, grape, and broccoli, *R. canina* NVs display a distinctive bioactive profile characterized by a high content of phenolic and flavonoid compounds. This enrichment underlies interesting antioxidant and anti-inflammatory activities, distinguishing them from the lipid- or terpenoid-rich EVs of ginger and citrus. On the other hand, the composition of *R. canina* NVs resembles that of green tea EVs, one of the most recognized natural antioxidants rich in catechins and gallic acid [22,52]. Concerning antioxidant properties, *R. canina* NVs exhibited strong radical scavenging and metal-reducing capacities, as demonstrated by in vitro assays (Table 2). The total polyphenol content (61.16 ± 4.36 mg GAE/particles) and total flavonoid content (2.69 ± 0.18 mg RE/particles) highlight the richness in bioactive constituents. Radical scavenging activity assessed by DPPH (56.57 ± 4.44 mg TE/particles) and ABTS (76.32 ± 3.58 mg TE/particles) assays confirmthe ability of NVs to counteract both lipophilic and hydrophilic radicals. Their effectiveness in reducing metal ions was supported by high FRAP (56.10 ± 1.48 mg TE/particles) and CUPRAC (97.48 ± 1.17 mg TE/particles) values, with the latter indicating a particularly strong overall antioxidant potential. Moreover, the metal-chelating capacity (24.53 ± 3.70 mg EDTAE/particles) suggests an additional protective mechanism against the formation of reactive oxygen species [65]. These antioxidant capacities are comparable to or even higher than those reported for grape NVs, which are also polyphenol-rich, and for broccoli NVs, known for their glucosinolate content and redox-modulating activity [66,67]. Beyond antioxidant properties, *R. canina* NVs demonstrated significant enzyme inhibitory activity relevant to neurodegeneration, skin diseases, and carbohydrate metabolism (Table 3). Moderate inhibition of acetylcholinesterase (AChE; 0.65 ± 0.08 mg GALAE/particles) and more pronounced inhibition of butyrylcholinesterase (BChE; 3.50 ± 0.05 mg GALAE/particles) suggest a potential role in supporting neurotransmitter function, particularly in the later stages of Alzheimer’s disease where BChE becomes predominant [68]. Compared to other natural cholinesterase inhibitors, such as huperzine A, the observed values indicate a moderate activity that could be biologically relevant, especially in combination with other neuroprotective compounds [69]. The strong inhibition of tyrosinase (59.56 ± 3.35 mg KAE/particles) indicates possible applications for managing skin hyperpigmentation. This value is comparable to other plant-derived inhibitors, such as kojic acid and certain flavonoids, supporting the potential use of NVs in topical formulations for controlling melanin synthesis [70]. Lastly, the inhibition of α-glucosidase (1.21 ± 0.02 mmol ACAE/particles) and α-amylase (0.22 ± 0.03 mmol ACAE/particles) highlights a potential hypoglycemic effect, relevant to postprandial glucose management in type 2 diabetes [71]. In particular, the inhibition of α-glucosidase observed for *R. canina* NVs is biologically relevant and comparable to values reported for other natural sources expressed in acarbose equivalents. α-Glucosidase catalyzes the terminal step of carbohydrate digestion, and its inhibition delays glucose absorption, thereby contributing to the attenuation of post-prandial hyperglycemia. Comparable levels of activity have been reported for several plant extracts. For instance, the dichloromethane extract of *Malabaila lasiocarpa* exhibited 0.69 ± 0.02 mmol ACAE/particles, while acorn flour extracts reached approximately 0.99 mmol ACAE/particles. These values are slightly lower than those observed for *R. canina* NVs, indicating a comparable or superior inhibitory capacity. Moreover, *Ricinodendron heudelotii* extracts displayed values around 1.3 ± 0.0 mmol ACAE/particles, comparable to the activity found for *R. canina* NVs [34,72,73]. The biological activity of *R. canina* NVs was further confirmed through cellular studies. Cytotoxicity evaluation on THP-1 monocytes revealed that the NVs are biocompatible, even at high concentrations, with no toxic effects observed (Figure 2a). Furthermore, *R. canina* NVs significantly improved cell viability under oxidative stress induced by H_2_O_2_, indicating strong cytoprotective and antioxidant activity at the cellular level (Figure 2b). This effect is likely attributable to the polyphenol-rich composition of the NVs, which can both scavenge reactive oxygen species and activate endogenous defense mechanisms. In terms of anti-inflammatory potential, *R. canina* NVs significantly reduced the production of pro-inflammatory cytokines IL-1β, IL-8 and IL-6 together with TNF cytochine in parallel with a reduction in NF-κB activation, indicating a coordinated interference with the cytokine/NF-κB axis in LPS-stimulated THP-1 monocytes (Figure 2c). This effect is consistent with what has been reported for lemon-derived vesicles, which suppressed ERK1/2–NF-κB signaling and reduced IL-1β, IL-6, and TNF in LPS-stimulated macrophages, thereby attenuating the inflammatory response. A similar multi-cytokine down-modulation has been reported for ginger-derived EVs, which inhibit IL-1β, IL-6, and TNF and dampen NF-κB activity in myeloid cells and in vivo models of inflammation. The effect observed in our system is also consistent with the known pharmacology of *Rosa* spp. and with the flavonoid-rich cargo typically found in rose hips; in fact, flavonoids are well-documented inhibitors of NF-κB-induced transcription and IL-1β, IL-6, and TNF production, providing a biologically plausible explanation for the broad cytokine attenuation observed in this study [74,75,76,77,78]. Accumulating evidence suggests that plant-derived nanovesicles may play a significant role in shaping the gut microbiota and further exert effects on human health [79]. Moreover, a recent study proved that lemon-exosome-like nanoparticles increased the bile resistance of probiotic strain *L. rhamnosus* GG (LGG) [80]. Therefore, this preliminary work investigated the potential of plant NVs to protect gut microbiota during the antibiotic stress using in vitro gastrointestinal simulation system GIS1. Despite their widespread use, in vitro gastrointestinal models present several inherent limitations, especially as they lack the full physiological complexity of the human digestive system, including peristaltic motion, mucus interactions, and host immune responses. However, validating in vitro findings enhances predictive reliability in vivo studies. Moreover, the adoption of standardized protocols promotes reproducibility and comparability across studies. The selected antibiotic, kanamycin, is an aminoglycoside antibiotic that specifically targets the 30S ribosomal subunit in bacteria, interfering with the protein synthesis [81]. While its primary antimicrobial mechanism is inhibiting protein synthesis, it can also exhibit a pro-oxidant effect that promotes the formation of reactive oxygen species in the bacterial cells and contributes to its antibacterial effect [82,83]. On the other hand, *R. canina* NVs demonstrated significant antioxidant properties and is rich in polyphenols like naringin that proved to be gut microbiota modulators [76,84]. In our experiments, after 5 days of kanamycin treatment, the total microbial load of culturable bacteria decreased by log 5.67 CFU/mL compared to the control experiment (M), while the co-administration of antibiotics and NVs lowers the total counts by log 1.13 CFU/mL. That significant difference could be related to the *R. canina* NVs protective role in stress conditions, as the nanovesicles supplementation (MEVs treatment) did not significantly change the aerobic and anaerobic counts. However, more research is needed to understand the molecular mechanism underlying the protective effect of *R. canina* NVs on microbes from human intestine. Still, some studies have demonstrated the uptake of plant-derived nanovesicles by gut bacteria and their effect on microbial metabolism [85,86]. A good example of this is the ginger-derived exosome-like nanoparticles that can modulate the tryptophan metabolism of gut Lactobacillus and alleviate colitis in the mouse model [85].

Reports have consistently revealed that the abundance of Lactobacillus and other beneficial gut bacteria like Bifidobacterium decreased under antibiotic treatment, while the antimicrobials can potentially promote the growth of other bacteria like Enterococcus and Clostridium [49]. In this study, both plate-based and qPCR results proved that the antibiotic lowered both beneficial bacteria density (up to 5 log/mL), mostly lactobacilli. The administration of *Rosa* nanovesicles and kanamycin (MKmEVs treatment) partially protected the population of Lactobacillus, which decreased by 1.22 log/mL compared to the control experiment (M). Conversely, the antimicrobial effect of kanamycin on *Bifidobacterium* spp. was significantly reduced by *R. canina* NVs and the qPCR counts in M and MKmEVs experiments were similar (approximately log 8.6 log/mL). The major shift in the counts of beneficial bacteria after antibiotic exposure was not a surprise, compared to the slow decrease in the abundance of beneficial microbes noted after NVs administration. Thus, a higher decline was observed for Bifidobacterium population (0.41 log/mL) compared to Lactobacillus that lower by 0.28 log/mL in MEVs experiments. Some reports have shown that plant nanoparticles and nanovesicles could have different effects on specific gut bacterial strains growth. However, in our study, the Lactobacillus population slowly decreased in the presence of *R. canina* NVs, while ginger and lemon nanoparticles were reported to enhance lactobacilli. This different behavior could be related to the peculiar biochemical composition of *R. canina* NVs, which are particularly enriched in phenolic compounds such as quercetin, catechins, and gallic acid. These molecules, although known for their antioxidant and anti-inflammatory properties, can also exert selective antimicrobial or bacteriostatic effects on certain Gram-positive taxa, including Lactobacillus species, depending on their concentration and structural form. Therefore, the observed reduction may reflect a modulatory rather than a detrimental effect, consistent with the general capacity of plant polyphenols to transiently reshape gut microbial communities [80,85,87]. Still, results are difficult to compare as there are differences in the composition of plant nanovesicles, as well as in the experimental conditions. Thus, plant-derived exosome-like nanoparticles from ginger increased Lactobacillaceae and Bacteroidales S24-7, had no effect on Bacteroides fragilis and Escherichia coli growth, inhibited Ruminococcaceae growth, and decreased Clostridiaceae [85]. The research demonstrated that the content of ginger-derived nanoparticles, especially the phosphalidic acid lipids, are signals for preferential uptake by probiotic strain *Lactobacillus rhamnosus* GG (LGG), while microRNAs from nanovesicles target bacterial genes [85]. Also, tartary buckwheat-derived nanovesicles were found to promote the growth of probiotic strain LGG [88]. The lemon-exosome-like nanoparticles were used as prebiotics to enhance Lactobacillus population, and they increased the bile resistance of LGG strain and inhibited the *Clostridioides difficilae* infection responsible for antibiotic-associated colitis [80].

The ability of *R. canina* NVs to minimize antibiotic-induced perturbation in gut microbiota was demonstrated by the clear decrease in the quantitative F/B ratio for MKm treatment compared to the other experiments. Moreover, the co-administration of both kanamycin and plant NVs (MKmEVs) reduced both populations of Firmicutes and Bacteroidetes, but at the end of the experiment, the quantitative ratio F/B is similar to the ratio from the control experiment. The antibiotic kanamycin is mostly used against Gram-negative bacteria but is known to be effective against some Gram-positive bacteria (e.g., Staphylococcus aureus and S. epidermidis), while having limited activity against other Gram-positive bacteria [81]. Some Lactobacillus species often exhibit intrinsic resistance to kanamycin [50]. The plant NVs uptake by various bacterial cells and their ability to metabolize plant polyphenols and reduce the antibiotic-induced oxidative stress could partially explain the capacity of *R. canina* NVs to minimize the kanamycin effects on intestinal microbiota. These results raised the possibility of using nanotherapy to protect human gut microbiota during the antibiotic administration, but more research is needed to find the practical application. Although the present study is limited to in vitro experiments, the results provide a solid basis for the subsequent in vivo validation of *R. canina* nanovesicles. Future investigations should clarify their mechanisms of action, bioavailability, and interactions within physiological systems to confirm their potential biological relevance.

## 4. Materials and Methods

### 4.1. Materials

*R. canina* dry berries were purchased from the local herbal shop. 2-(N-Morpholino)Ethanesulfonic Acid (MES), Calcium chloride (CaCl_2_), Sodium Chloride (NaCl), Methanol, and Ethanol were purchased from Sigma Aldrich. 3-(4,5-dimethylthiazol-2-yl)-2,5-diphenyltetrazolium bromide (MTT), Phosphate-Buffered Saline (PBS), Dimethyl Sulfoxide (DMSO), Glutaraldehyde (25%), 2,2-Diphenyl-1-picrylhydrazyl (DPPH), ABTS radical cation (2,2′-azino-bis(3-ethylbenzothiazoline)-6-sulphonic acid), TPTZ (2,4,6-tri(2-pyridyl)-s-triazine), FeCl_3_, Cu(II), neucoproine, and Trolox were purchased from Sigma-Aldrich (St. Louis, MO, USA). Human monocytic cell line THP-1 (ATCC TIB-202) was purchased from American Type Culture Collection (ATCC) (Manassas, VA, USA). The cells were cultured under standard conditions. RPMI 1640 medium (RPMI), fetal bovine serum (FBS), trypsin, and penicillin/streptomycin were purchased from Euroclone (Pero, MI, Italy). All the other chemicals were of analytical grade and were obtained from Sigma-Aldrich unless otherwise indicated. Seahorse XF Glycolytic Rate Assay Kit, XF DMEM medium, XF glutamine, XF pyruvate, and XF glucose were purchased from Agilent Technologies (Santa Clara, CA, USA).

### 4.2. Methods

#### 4.2.1. Isolation Procedure for *R. canina* Berries Nanovesicles

To isolate *R. canina* NVs, 5 g of dried berries were weighed and rehydrated in 10 mL of VIB extraction buffer (composed of 20 mM MES, 2 mM CaCl_2_, and 0.1 M NaCl; pH adjusted to 6.0). The mixture was gently agitated at 4 °C overnight to ensure complete rehydration of the plant matrix. The vesicle isolation was subsequently conducted by means of differential ultracentrifugation (dUC) with Beckman L80 XP ultracentrifuge (Beckman Coulter, CA, USA) [89]. An initial low-speed centrifugation step was performed at 700× *g* for 20 min at 4 °C to remove contaminant debris. The supernatant was then passed through a 0.45 µm pore-size filter (Millex^®^ 0.45 μm from Merck Millipore Inc., Billerica, MA, USA) to eliminate residual particulates and underwent a series of centrifugation steps at progressively higher speeds: first, at 10,000× *g* for 60 min and then, at 100,000× *g* for an additional 60 min.

The resulting pellet, enriched in nanovesicles, was resuspended in different solvents depending on downstream applications: 100 µL of 100% ethanol for antioxidant evaluations, 200 µL of phosphate-buffered saline (PBS) containing 10% dimethyl sulfoxide (DMSO) for cellular assays, and 200 µL of 100% methanol for LC-QTOF-MS metabolomic analysis. Moreover, *R. canina*-NVs was also purified by a density gradient [90]. Briefly, 10 mL of *R. canina* were diluted 1:2 (*v*/*v*) in phosphate-buffered saline (PBS) and sequentially centrifuged at 1200× *g* for 20 min, three times at 3000× *g* for 20 min, and finally at 10,000× *g* for 60 min, all at 4 °C. The resulting supernatant was filtered through a 0.45 µm pore-size membrane and subjected to ultracentrifugation using a Type 70.1 Ti rotor (Beckman Coulter, Indianapolis, IN, USA) at 100,000× *g* for 90 min at 4 °C. The obtained pellet was resuspended in 10 mM Tris-HCl (pH 8.6) and further purified by density gradient ultracentrifugation on iodixanol (OptiPrep™, Sigma-Aldrich, Burlington, MA, USA). A discontinuous gradient was prepared using 1.5 mL layers of 50%, 30%, and 10% (*w*/*v*) iodixanol solutions in 10 mM Tris-HCl (pH 8.6), onto which the NV suspension was carefully loaded.

#### 4.2.2. Nanoparticle Tracking Analysis (NTA) and Scanning Electron Microscopy (SEM)

The concentration and size distribution of the *R. canina* NVs pool population and *R. canina*-NVs fractions obtained from density gradient purification were assessed using nanoparticle tracking analysis (NTA) with a NanoSight NS300 instrument (Malvern Panalytical, Malvern, UK). Samples were diluted in sterile, 0.22 µm-filtered phosphate-buffered saline (PBS) to reach the optimal particle concentration for analysis [21]. Each sample was subjected to five independent measurements in two separate experimental replicates. For scanning electron microscopy (SEM), vesicles were initially resuspended in 50 µL of 0.22 µm-filtered PBS. Protein content was determined using the Bradford assay [91], following the manufacturer’s instructions. A volume corresponding to 2 µg of total protein was diluted in 2 mL of 2.5% glutaraldehyde solution (*v*/*v* in 1× PBS) and incubated for 15 min at room temperature to promote fixation. After fixation, the suspension was diluted with 15 mL of double-distilled water (filtered through a 0.22 µm membrane) and transferred into Vivaspin centrifugal concentrators (molecular weight cut-off: 300 kDa). The samples were centrifuged at 3000× *g* for 3 min, and the eluate was discarded. Two additional washing steps with 10 mL of ultrapure water were performed under the same centrifugation conditions. For SEM preparation, vesicle suspensions were diluted at 1:500 and 1:1000 ratios, and 20 µL of each dilution was applied to 12 mm glass coverslips. Following adhesion, the samples were fixed and sputter-coated with a thin conductive layer prior to imaging.

#### 4.2.3. Dynamic Light Scattering (DLS)

The Zeta-potential of *R. canina* NVs was determined using the Nicomp^®^ Nano N3000 Dynamic Light Scattering (DLS) system (Entegris, Billerica, MA, USA) [92]. This instrument allows for the measurement of the Zeta-potential and particle size with diameters ranging from 0.3 nm to 10 µm, combining DLS technology with Frequency and Phase Analysis Light Scattering (PALS) modes specific for Zeta-potential determination. The system is equipped with a high-power red laser diode (15–100 mW, 630 nm) and two detectors, PMT and APD, positioned at 90 degrees. The applicable electric field can be adjusted from 1 to 250 V/cm. The Nicomp algorithm also allows for the resolution of close multi-modal distributions with high resolution.

#### 4.2.4. Immunoblotting Analysis

To investigate the presence of plant vesicular marker TET8 in *R. canina* NVs isolated via density gradient centrifugation, 10 μg of total protein of NVs or *R. canina* crude extract were mixed with 5× sample buffer (1 M Tris-HCl pH 6.8, 5% SDS, 6% glycerol, 0.01% bromophenol blue) supplemented with 125 mM DTT. Samples were heated at 95 °C for 5 min and subjected to SDS-PAGE on 10% acrylamide gels. Gels were transferred onto polyvinylidene difluoride (PVDF) membranes using the Trans-Blot Turbo Transfer System (Bio-Rad, Hercules, CA, USA). After blocking, membranes were incubated overnight at 4 °C with a primary antibody against TET8 from PhytoAB, (San Jose, CA, USA) and Calnexin from Santa Cruz Biotechnology (Santa Cruz, CA, USA). Detection was performed using HRP-conjugated secondary antibodies (Cell Signaling Technology, Beverly, MA, USA) and visualized via enhanced chemiluminescence (ECL) (GE Biosciences, Piscataway, NJ, USA).

#### 4.2.5. Antioxidant In Vitro Assays

The total phenolic content (TPC) was determined using the Folin–Ciocalteu method [44]. Briefly, an aliquot of the NV suspension was mixed with the Folin–Ciocalteu reagent and sodium carbonate solution. After incubation in the dark at room temperature, the absorbance was measured at 765 nm using an Infinite^®^ Tecan plate reader (Tecan Group Ltd., Männedorf, Switzerland). Results were expressed as gallic acid equivalents (GAEs) per gram of sample. The total flavonoid content (TFC) was assessed via the aluminum chloride colorimetric method. NVs were mixed with a solution of aluminum chloride in methanol. After incubation, absorbance was recorded at 415 nm, and results were expressed as rutin equivalents (REs) per gram of sample. For the evaluation of radical scavenging activity, both DPPH and ABTS assays were employed [93]. In the DPPH assay, NVs were mixed with a freshly prepared methanolic DPPH solution and incubated in the dark at room temperature. 0.1 mM DPPH was dissolved in 100% methanol. Absorbance was measured at 517 nm. In the ABTS assay, ABTS radicals with a starting concentration of 7 mM were generated by reaction with 2.45 mM of potassium persulfate, and the resulting solution was diluted to an absorbance of 0.700 ± 0.020 at 734 nm. The NVs were then incubated with the diluted ABTS^+^ solution, and absorbance was measured at 734 nm. In both assays, scavenging activity was calculated based on the decrease in absorbance compared to a blank control. The ferric-reducing antioxidant power (FRAP) was measured using a reagent composed of acetate buffer, TPTZ (2,4,6-tri(2-pyridyl)-s-triazine) solution in HCl, and ferric chloride [94]. NVs were added to the FRAP reagent, incubated in the dark, and absorbance was measured at 593 nm. The cupric reducing antioxidant capacity (CUPRAC) assay was conducted by mixing the NVs with a solution of copper(II) chloride, neocuproine in ethanol, and ammonium acetate buffer (pH 7) [94]. After incubation in the dark, absorbance was measured at 450 nm. The radical scavenging and reducing power abilities were expressed as the equivalent of trolox (mg TE/g). The metal-chelating activity was evaluated using the ferrous ion chelation assay. NVs were incubated with FeCl_2_ solution, followed by the addition of ferrozine reagent. After incubation, the absorbance was measured at 562 nm. The decrease in the formation of the ferrozine-Fe^2+^ complex was used to estimate the chelating capacity. EDTA was used as a positive control in the metal-chelating assay, and the results are explained as the EDTA equivalent (mg EDTAE/g). For each assay a specific internal standard was used: gallic acid for TPC, rutin for TFC; trolox for DPPH, ABTS, FRAP, and CUPRAC assays; and EDTA for metal-chelating activity.

The inhibitory activities of *R. canina* NVs against AChE, BChE, tyrosinase, α-amylase, and α-glucosidase were evaluated in 96-well microplates using previously reported methods with slight modifications [95]. Sample solutions were incubated with the respective enzyme and substrate, and blanks without enzyme were included. Absorbances were measured after incubation at the appropriate wavelength: 405 nm (AChE/BChE), 492 nm (tyrosinase), 630 nm (α-amylase), and 400 nm (α-glucosidase). Inhibitory activities were expressed as galantamine equivalents (mg GALAE/g) for AChE/BChE, kojic acid equivalents (mg KAEs/g) for tyrosinase, and acarbose equivalents (mg ACAEs/g) for α-amylase and α-glucosidase. All measurements were performed in triplicate.

#### 4.2.6. Untargeted Analysis of Polyphenol Content by Q-TOF LC/MS Mass Spectrometry

To extract polyphenolic compounds from *R. canina* NVs, 40 µL of an extraction mixture consisting of 70% methanol acidified with 3% formic acid was added to each sample. The mixture was then vortexed for 10 min using a thermomixer to facilitate compound solubilization. Following mixing, samples were centrifuged at 10,000× *g* for 10 min at 4 °C. The supernatant, containing the solubilized polyphenols, was carefully transferred into glass vials for subsequent chromatographic analysis. An untargeted metabolomic approach was employed to profile the polyphenolic content using an Agilent 1260 Infinity II ultra-high-performance liquid chromatography (UHPLC) system coupled to an Agilent 6530 quadrupole time-of-flight (Q-TOF) mass spectrometer. The instrument was equipped with an Agilent JetStream electrospray ionization (ESI) source. Separation was achieved using a Waters Acquity XBridge BEH Amide C18 column (150 mm × 2.1 mm, 1.7 µm particle size) (Waters Corporation, Milford, MA, USA), maintained at a controlled temperature between 25 and 30 °C. The mobile phases consisted of water (solvent A) and acetonitrile (solvent B), both containing 0.2% formic acid, delivered at a flow rate of 0.35 mL/min. The chromatographic gradient was designed as follows: solvent B started at 5% and increased linearly to 45% over 15 min; from 15 to 18 min, the proportion of B rose to 95%, which was maintained for an additional 2 min. The gradient then returned to the initial condition (5% B) at 20.1 min, and the run concluded at 23 min. Mass spectrometric data were acquired in both positive and negative ionization modes. The Q-TOF was operated under the following settings: capillary voltage 3500 V, drying gas temperature 250 °C, sheath gas temperature 300 °C, nebulizer pressure 35 psi, and sheath gas flow rate of 12 L/min. Data-dependent acquisition was performed over a mass range of *m*/*z* 40–1700 for both MS and MS/MS analyses, applying a collision energy of 30 V. Raw mass spectrometry data were processed using Agilent MassHunter Qualitative Analysis software and MS-DIAL (version 4.9) for peak picking and tentative compound identification [21]. Due to the absence of internal standards, the relative quantification of detected polyphenols was based on their proportional contribution to the total phenolic content (TPC), as determined spectrophotometrically. Each analysis was carried out in three independent biological replicates.

#### 4.2.7. Cell Treatments

The human monocytic cell line THP-1 (ATCC^®^ TIB-202™, Manassas, VA, USA) was employed as an in vitro model to assess the biological effects of *R. canina* NVs. Cells were cultured in an RPMI-1640 medium (Euroclone S.p.A., Pero, Italy), supplemented with 10% (*v*/*v*) heat-inactivated fetal bovine serum (FBS), 2 mM L-glutamine, 100 U/mL penicillin, and 100 µg/mL streptomycin. Cultures were maintained at 37 °C in a humidified incubator with 5% CO_2_. Prior to treatment, THP-1 monocytes were differentiated into macrophage-like cells by exposure to 100 nM phorbol 12-myristate 13-acetate (PMA) for 48 h. To evaluate the cellular uptake of *R. canina*-derived NVs, THP-1 cells were incubated with NVs previously labeled with 1,1′-Dioctadecyl-3,3,3′,3′-Tetramethylindocarbocyanine Perchlorate (DiL; Thermo Fisher Scientific, Carlsbad, CA, USA) and analyzed by fluorescence microscopy [96]. For vesicle labeling, 50 µM DiL was added to the supernatant obtained after centrifugation at 12,000× *g*, while PBS processed in parallel was used as a negative control. After 30 min of incubation at room temperature, the excess dye was removed by washing the NVs with PBS through ultracentrifugation at 40,000× *g* for 70 min at 4 °C. For the uptake assay, THP-1 cells (5 × 10^4^) were exposed to the DiL-labeled NVs for 1 h, fixed with 4% paraformaldehyde for 20 min, and stained with FITC-conjugated phalloidin for 30 min to visualize F-actin filaments. Nuclei were counterstained using VECTASHIELD^®^ Vibrance Antifade Mounting Medium containing DAPI. Fluorescence images were acquired with a Nikon Eclipse TE2000-S fluorescence microscope (Nikon, Tokyo, Japan) equipped with an F-View II FireWire camera (Olympus Soft Imaging Solutions) and analyzed using CellF Imaging Software (Olympus Soft Imaging Solutions) [97,98].

For viability and antioxidant and anti-inflammatory assays, cells were then washed and allowed to rest for 24 h in fresh medium before experimental procedures. Cell viability upon treatment with NVs was evaluated via the MTT [3-(4,5-dimethylthiazol-2-yl)-2,5-diphenyltetrazolium bromide] assay [21]. Differentiated THP-1 cells were seeded into 96-well plates at a density of 1 × 10^4^ cells/well in 100 µL of complete RPMI medium. After 24 h, cells were treated with increasing concentrations of NVs resuspended in PBS containing 10% DMSO. Vehicle controls (PBS + DMSO) were included at equivalent dilutions. Following 24 h of treatment, 10 µL of MTT solution (5 mg/mL in PBS) was added to each well and incubated for 3 h at 37 °C. The resulting formazan crystals were solubilized using 100 µL of DMSO, and absorbance was measured at 570 nm using a Tecan Infinite^®^ microplate reader. Cell viability was expressed as a percentage relative to untreated control cells.

To simulate oxidative stress, cells were exposed to hydrogen peroxide (H_2_O_2_, final concentration 100 and 250 μM) for an additional 24 h with and without NVs treatment. After another 245 h, 10 µL of MTT solution (5 mg/mL) was added to each well and incubated for 3 h at 37 °C [21]. After removing the medium, formazan crystals were dissolved with 100 µL of DMSO and absorbance was measured at 570 nm using a Tecan Infinite^®^ spectrophotometer. Also, in this case, cell viability was calculated as the percentage absorbance of treated samples relative to untreated controls. The protective effect of *R. canina* NVs was deduced from the increased viability of cells exposed to both EVs and H_2_O_2_, compared to cells treated with H_2_O_2_ alone. Finally, gene expression analysis was performed to assess the pro- or anti-inflammatory effects of NVs. THP-1-derived macrophages were treated for 24 h with NVs, followed by stimulation with lipopolysaccharide (LPS, 100 ng/mL) for 4 h to induce cytokine expression. Total RNA was isolated using TRIzol, according to the manufacturer’s instructions. Reverse transcription was conducted using the Maxima H Minus First Strand cDNA Synthesis Kit (Thermo Scientific), and qRT-PCR was carried out SYBR Green Master Mix (Bio-Rad, Hercules, CA, USA) following the manufacturer’s protocol using a StepOnePlus thermocycler (Applied Biosystems, Foster City, CA, USA) [8]. Specific primers were used to amplify human IL-8, IL-1β, IL-6, p-NF-κB, TNF-α, and GAPDH as the housekeeping gene (Table S1 reports primer sequences). Relative gene expression was calculated using the 2^−ΔΔCt^ method, and results were expressed as fold changes relative to the untreated control.

#### 4.2.8. In Vitro Simulation Using the GIS1 System

Tests were conducted using an in vitro gastrointestinal simulator (GIS1 system, www.gissystems.ro e.g., accessed on 5 May 2025) to simulate the human colon environment. The microbiota sample kept in the Microbiota Culture Collection—ColHumB Registration number: RBFB6N18—consisted of a mix of microbiota obtained from several adult donors of both sexes who had not been treated with antibiotics or any other interfering drugs over the past six months, as these may alter the normal microbiota. The sample was previously collected from coproculture, mixed to obtain a representative microbial community, analyzed, and stored in frozen 10% glycerol until needed. The reconstitution process followed the protocol previously described and was performed with a mean interval of 7–10 days to achieve a consistent microbial profile [46]. The microbiota was grown under controlled microaerophilic conditions, at a constant temperature of 37 °C with the pH maintained at a value of 6, while their evolution was monitored during the simulation process. Four experiment setups were performed: (1) the control experiment (M) with added sterile distilled water; (2) the MKm experiment with kanamycin treatment; (3) the MEVs experiment with NVs administration; (4) the MKmEVs experiment with both antibiotic and NVs supplementation. In two experiments (MEVs, MKmEVs), the microbiota were treated daily with 0.5 mL of NVs solution, containing 20.1 × 10^9^ particles/mL that were directly added to the simulated environment under sterile conditions. Kanamycin sulfate (Sigma-Aldrich, St. Louis, MO, USA) was used to induce antibiotic-driven dysbiosis at a final concentration of 2 mg/mL in the simulation medium as previously described [46,47]. At the end of the simulation (day 5), samples were collected and centrifugated at 4000× *g* for 15 min. The sediment was microbiologically analyzed within 24 h or preserved in glycerol 20% for qPCR analysis. Three biological replicates were performed for each experimental condition. Statistical analysis was conducted using the IBM SPSS Statistics 23 software package (IBM Corporation, Armonk, NY, USA).

#### 4.2.9. Microbiome Analysis Using Microbiological Methods and the qPCR Technique

For microbiological analysis, samples were diluted and then plated as solutions onto media. The selective media used were MRS agar (Oxoid, Thermo Fisher Scientific, Waltham, MA, USA) for lactic acid bacteria, BSM agar for bifidobacteria, and Nutrient agar (Oxoid, Thermo Fisher Scientific, Waltham, MA, USA) for aerobes and anaerobes counts, respectively. Plates were incubated for 24–48 h at 37 °C for aerobic counts, while plates with nutrient broth agar for anaerobic counts were placed in a standard anaerobic jar.

Genomic DNA from samples was extracted using the Quick-DNA Miniprep Plus kit (Zymo Research, Irvine, CA, USA) according to the manufacturer’s instructions for bacteria, and DNA concentrations were measured with a NanoDrop 8000 (Thermo Fisher Scientific, Waltham, MA, USA). All qPCR reactions were performed as previously described using a Rotor-Gene 6000 5plex HRM (Qiagen-Corbett Life Science, Sidney, Australia) instrument and software to generate the standard curve and microbial quantification [99]. Standard curves were routinely performed for each qPCR using serial dilutions of control DNA and primers selected for highly conserved regions of genes [100,101]. Thus, a mix of *Lactobacillus acidophilus*, *L. plantarum*, and *L. rhamnosus* (primer set Lac-1F/Lac-2R) was used for *Lactobacillus* spp. and Firmicutes (primer set Firm-934F/Firm-1060R) quantification (R2 = 0.9918) and a mix of Bifidobacterium animalis and B. bifidum for Bifidobacterium sp. (primer set g-BIFID-F/g-BIFID-R) quantification (R^2^ = 0.9995). A strain of Bacteroides fragilis ATCC 25,285 was used for Bacteroidetes phylum (primer set Bac-960F/Bac-1100R) quantification (R^2^ = 0.9682). The final volume in all reactions was 25 μL, including 1 μL of template DNA, 12.5 μL of Maxima SYBR Green Mix (Thermo Fisher Scientific) (Waltham, MA, USA), and 0.5 μL of each primer. The cycling parameters for the 3-step melting process were 10 min at 95 °C, followed by 40 cycles for 15 s at 95 °C, 30 s at 60 °C, and 30 s or 45 s at 72 °C, based on the PCR product size. The specificity of qPCR reactions was confirmed by melting curve analysis. Reactions were carried out in triplicate, and the results were statistically analyzed.

## 5. Conclusions

The present study outlined a systematic analysis of *R. canina*-derived nanovesicles, highlighting their validity as natural carriers of bioactive compounds. The integrated approach through morphological, biochemical, and functional analyses confirmed the structural integrity of NVs and their peculiar polyphenolic and flavonoid chemical composition, as well as the fact that promising bioactive profile *R. canina* NVs were particularly rich in (-)-epicatechin, quercetin-3-*O*-rutinoside, and gallic acid, metabolites characterized by documented antioxidant and anti-inflammatory activities. The high capacity to neutralize free radicals, reduce metal ions, and chelate reactive metals, as detected in DPPH, ABTS, FRAP, and CUPRAC tests, underlines the potential of NVs as broad-spectrum antioxidant agents, suggesting a rationale for nutraceutical applications. In vitro studies on human THP-1 monocytes have also confirmed the absence of cytotoxicity and a significant cytoprotective and anti-inflammatory effect, with a remarkable reduction in pro-inflammatory cytokines. The capacity of *Rosa canina* NVs to minimize the kanamycin effects on intestinal microbiota raised the possibility to use these small carriers to protect human gut microbiota during antibiotic administration. Overall, the evidence positions *R. canina* NVs as a treasure trove of antioxidant molecules with potential implications in the pharmacological and nutraceutical fields.

## Figures and Tables

**Table 1:** Identification of polyphenol species in *R. canina* NVs through LC/MS. The concentration is reported as µg of compound detected in samples obtained from 4.9 × 10^11^ particles, corresponding to the NV fraction isolated from 5 g of *R. canina* berries. For the most abundant metabolites, the chromatogram and the related *m*/*z* ratio were reported in Figure S3.

Identified Metabolite in *R. canina* NVs	Concentration (µg/4.9 × 10^11^ Particles)
(-)-epicatechin	6.37 ± 0.42
Hederagenin glucoside	2.00 ± 0.17
Phloretin-2′-*O*-glucoside	1.99 ± 0.15
Quercetin-3-*O*-rutinoside	1.95 ± 0.36
Gallic acid	1.10 ± 0.24
Naringenin-7-*O*-glucoside	0.76 ± 0.12
Naringenin	0.52 ± 0.05
Eriodictyol-7-*O*-glucoside	0.49 ± 0.04
6-(3-Benzoyloxy-2-hydroxypropoxy)-3,4,5-trihydroxyoxane-2-carboxylic acid	0.49 ± 0.06
1-*O*-Galloyl-6-*O*-cinnamoylglucose	0.47 ± 0.16
Procyanidin C1	0.35 ± 0.12
Procyanidin B1	0.33 ± 0.03
Gallic acid hexoside; PlaSMA ID-747	0.19 ± 0.04
Liquiritin	0.18 ± 0.04
*trans*-4-Coumaric acid	0.15 ± 0.01
Tormentic acid	0.12 ± 0.05
rhein	0.07 ± 0.02
epicatechin gallate	0.06 ± 0.02
Eriodictyol	0.06 ± 0.01
Methyl gallate	0.05 ± 0.01
Salicylic acid	0.03 ± 0.01
Isoquercitrin	0.03 ± 0.01
Corosolic acid	0.02 ± 0.01

**Table 2:** Antioxidant and metal-chelating activity of *R. canina* NVs. Results were expressed as mg/particles (9.8 × 10^10^) from 1 g of starting berries, and the values expressed are means ± SD of three parallel measurements. Analyses were performed using the following reference standards: gallic acid for total phenolic content (TPC); rutin for total flavonoid content (TFC); trolox for DPPH, ABTS, FRAP, and CUPRAC assays; and EDTA for metal-chelating activity.

TPC	TFC	DPPH	ABTS	FRAP	CUPRAC	Metal Chelating
mg GAE/Particles	mg RE/Particles	mg TE/Particles				mg EDTAE/Particles
61.16 ± 4.36	2.69 ± 0.18	56.57 ± 4.44	76.32 ± 3.58	56.10 ± 1.48	97.48 ± 1.17	24.53 ± 3.70

**Table 3:** Enzymatic inhibition capacity of *R. canina* NVs. Results were expressed as mg/particles (9.8 × 10^10^) from 1 g of starting berries and values expressed are means ± SD of three parallel measurements. Analyses were performed using the following reference standards: galantamine for AChE and BChE, kojic acid for tyrosinase, and acarbose for amylase and glucosidase inhibition.

AChE Inhibition	BChE Inhibition	Tyrosinase Inhibition	Amylase Inhibition	Glucosidase Inhibition
mg GALAE/Particles		mg KAE/Particles	mmol ACAE/Particles	
0.65 ± 0.08	3.50 ± 0.05	59.56 ± 3.35	0.22 ± 0.03	1.21 ± 0.02

**Table 4:** Effect of the *R. canina* NVs on the Firmicutes/Bacteroidetes (F/B) quantitative ratio in different experiments controls, MEVs Rosa with administration of *R. canina* NVs, MKm with kanamycin treatment, and MKmEVs Rosa with co-administration of *R. canina* NVs and kanamycin. Data are expressed as mean ± SD (n = 3).

Experiments	Firmicutes/Bacteroidetes (F/B) Quantitative Ratio
M	3.07
MEVs Rosa	2.95
MKm	2.39
MKmEVs Rosa	3.28

## Data Availability

The original contributions presented in the study are included in the article, further inquiries can be directed at the corresponding author.

## Supplementary Materials

The following supporting information can be downloaded at: https://www.mdpi.com/article/10.3390/ph18111672/s1, Figure S1: Analysis of *Rosa canina* NVs after density gradient separation. Evaluation of particle size and concentration of different density gradient fractions (from 2 to 10) is reported as Particles/mL obtained by NTA; Figure S2: Analysis of *Rosa canina* NVs through Dynamic Light Scattering (DLS) in order to evaluate the Zeta-potential. The zeta potential value was obtained after multiple analysis cycles for a total of 27 min of evaluation; Figure S3: LC/MS chromatograms of the main polyphenols detected. Peaks correspond to the compounds listed in the table: 1, (-)-epicatechin; 2, Gallic acid; 3, Hederagenin glucoside; 4, Phloretin-2′-*O*-glucoside; 5, Quercetin-3-*O*-rutinoside. Retention time (RT), mass accuracy (Diff, ppm), peak area, height, molecular formula, measured *m*/*z*, and theoretical mass are reported in the table; Table S1: Primer sequences.

## Abbreviations

The following abbreviations are used in this manuscript:

PDEVs	Plant-derived extracellular vesicles
NVs	Nanovesicles
ROS	Reactive Oxygen Species
NTA	Nanoparticles Tracking Analysis
SEM	Scanning Electron Microscopy
TPC	Total phenolic content
