# Supplementary material for: Nanovesicles from Rosa canina: A Treasure Trove of Antioxidant Potential for Oxidative Stress, Inflammation, and Gut Microbiota Modulation
Source: Pharmaceuticals (Basel). 2025 Nov 5;18(11):1672. doi: 10.3390/ph18111672 (PMC12655377; doi:10.3390/ph18111672)
Supplement: Supplementary file 1 [file pharmaceuticals-18-01672-s001.zip › pharmaceuticals-3932960-supplementary.pdf]

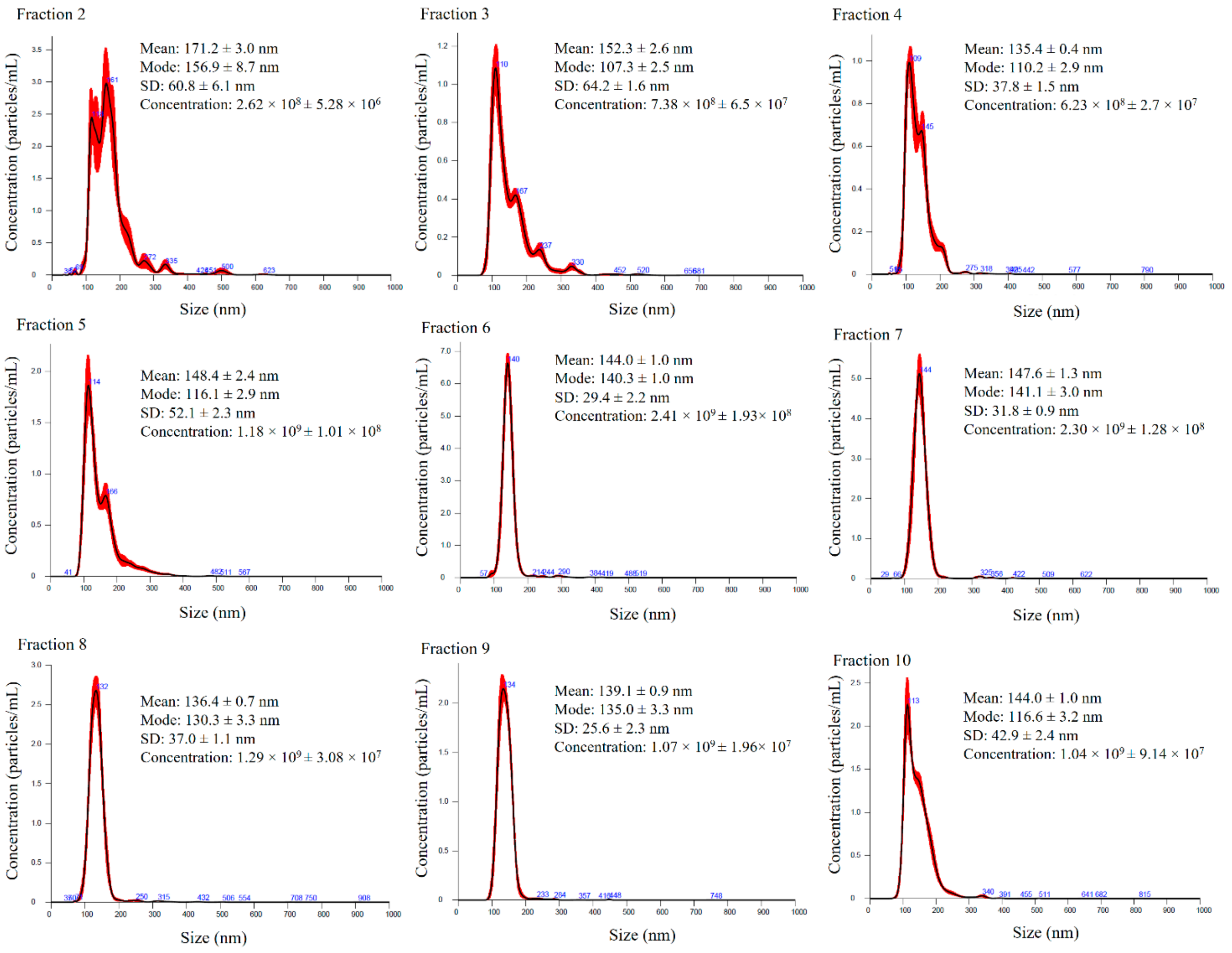

Figure S1. Analysis of *Rosa canina* NVs after density gradient separation. Evaluation of particle size and concentration of different density gradient fractions (from 2 to 10) is reported as Particles/mL obtained by NTA.

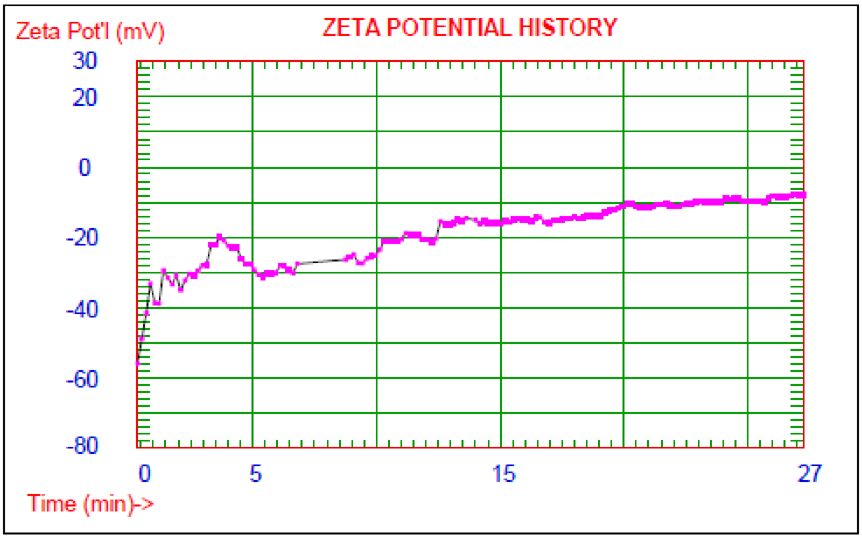

Sample Frequency:	----
Reference Frequency:	----
Cell Current:	14.13 mA (Cond.Factor = 0.70)
Avg. Phase Shift:	15.76 rad/sec.
Avg. Mobility:	-0.59 M.U.
Half-Width Mobility Dist.	----
Avg. Zeta Potential:	-7.93 mV
Half-Width Zeta Pot'l Dist.	----
Sample Temperature:	23 C
Liquid Viscosity:	0.933 cPoise
Index of Refraction:	1.333
Dielectric Constant:	78.500
Laser Wavelength:	635.0 nm
Scattering Angle:	-14.1 deg.
E-Field Strength:	6.00 V/CM
Channel Width:	20.0 uSec.
Run Time:	00:27:13

Figure S2. Analysis of *Rosa canina* NVs through Dynamic Light Scattering (DLS) in order to evaluate the Zeta-potential. The zeta potential value was obtained after multiple analysis cycles for a total of 27 minutes of evaluation.

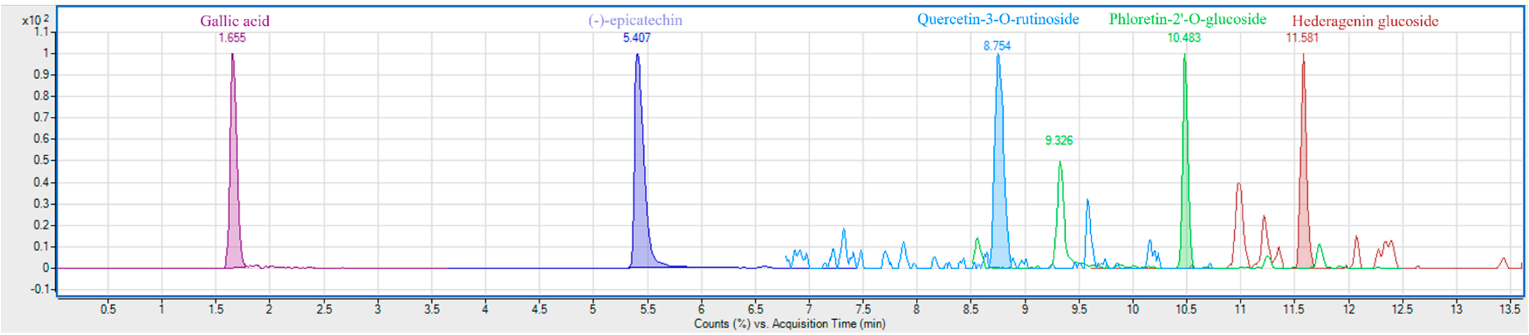

Cpd	Name	RT	Diff (DB, ppm)	Area	Height	Formula	m/z	Mass
1	(-)-epicatechin	5.407	-0.75	8506030	1362912	C15 H14 O6	289.072045	290.079256
2	Gallic acid	1.655	1.06	1668086	374997	C7 H6 O5	169.014119	170.021343
4	Phloretin-2'-O-glucoside	10.483	2.09	287126	78384	C21 H24 O10	435.12945	436.136037
3	Hederagenin glucoside	11.581	2.74	85142	22513	C36 H58 O11	711.394542	666.396087
5	Quercetin-3-O-rutinoside	8.754	7.44	60105	10434	C27 H30 O16	609.142106	610.148845

Figure S3. LC/MS chromatograms of the main polyphenols detected. Peaks correspond to the compounds listed in the table: 1, (-)-epicatechin; 2, Gallic acid; 3, Hederagenin glucoside; 4, Phloretin-2′-O-glucoside; 5, Quercetin-3-O-rutinoside. Retention time (RT), mass accuracy (Diff, ppm), peak area, height, molecular formula, measured m/z, and theoretical mass are reported in the table.

Table S1. Primer sequences

seq 5'-3'	name
TGCACCACCAACTGCTTAGC	GADPH F
GGCATGGACTGTGGTCATGAG	GADPH R
GCTCTCTTGGCAGCCTTCCT	IL-8 F
TTTCTGTGTTGGCGCAGTGT	Il-8 R
GTGGCAATGAGGATGACTTGTTC	IL-1β F
TAGTGGTGGTCGGAGATTCGTA	IL-1β R
GTGTTGCCTGCTGCCTTCC	IL-6 F
TCTGCCAGTGCCTCTTTGCT	IL-6 R
ACTCGCCACCCGGCTTCA	NF-kB F
CAGTGCCATCTGTGGTTGAAATACT	NF-kB R
ACTTTGGAGTGATCGGCCCC	TNF-α F
TGGGCTACAGGCTTGTCACT	TNF-α R
